# Broad neutralization of SARS-related viruses by human monoclonal antibodies

**DOI:** 10.1126/science.abc7424

**Published:** 2020-06-15

**Authors:** Anna Z. Wec, Daniel Wrapp, Andrew S. Herbert, Daniel P. Maurer, Denise Haslwanter, Mrunal Sakharkar, Rohit K. Jangra, M. Eugenia Dieterle, Asparouh Lilov, Deli Huang, Longping V. Tse, Nicole V. Johnson, Ching-Lin Hsieh, Nianshuang Wang, Juergen H. Nett, Elizabeth Champney, Irina Burnina, Michael Brown, Shu Lin, Melanie Sinclair, Carl Johnson, Sarat Pudi, Robert Bortz, Ariel S. Wirchnianski, Ethan Laudermilch, Catalina Florez, J. Maximilian Fels, Cecilia M. O’Brien, Barney S. Graham, David Nemazee, Dennis R. Burton, Ralph S. Baric, James E. Voss, Kartik Chandran, John M. Dye, Jason S. McLellan, Laura M. Walker

**Affiliations:** 1 Adimab LLC, Lebanon, NH 03766, USA.; 2 Department of Molecular Biosciences, The University of Texas at Austin, Austin, TX 78712, USA.; 3 U.S. Army Medical Research Institute of Infectious Diseases, Frederick, MD 21702, USA.; 4 Department of Microbiology and Immunology, Albert Einstein College of Medicine, New York, NY 10462, USA.; 5 Department of Immunology and Microbiology, The Scripps Research Institute, La Jolla, CA 92037, USA.; 6 Department of Epidemiology, The University of North Carolina at Chapel Hill, Chapel Hill, NC 27599, USA.; 7 Vaccine Research Center, National Institute of Allergy and Infectious Diseases, National Institutes of Health, Bethesda, MD 20892, USA.; 8 IAVI Neutralizing Antibody Center, The Scripps Research Institute, La Jolla, CA 92037, USA.; 9 Consortium for HIV/AIDS Vaccine Development (CHAVD), The Scripps Research Institute, La Jolla, CA 92037, USA.; 10 Ragon Institute of Massachusetts General Hospital, Massachusetts Institute of Technology, and Harvard, Cambridge, MA 02139, USA.; 11 Departments of Microbiology and Immunology, The University of North Carolina at Chapel Hill, Chapel Hill, NC 27599, USA.

## Abstract

Broadly protective vaccines against known and preemergent human coronaviruses (HCoVs) are urgently needed. To gain a deeper understanding of cross-neutralizing antibody responses, we mined the memory B cell repertoire of a convalescent severe acute respiratory syndrome (SARS) donor and identified 200 SARS coronavirus 2 (SARS-CoV-2) binding antibodies that target multiple conserved sites on the spike (S) protein. A large proportion of the non-neutralizing antibodies display high levels of somatic hypermutation and cross-react with circulating HCoVs, suggesting recall of preexisting memory B cells elicited by prior HCoV infections. Several antibodies potently cross-neutralize SARS-CoV, SARS-CoV-2, and the bat SARS-like virus WIV1 by blocking receptor attachment and inducing S1 shedding. These antibodies represent promising candidates for therapeutic intervention and reveal a target for the rational design of pan-sarbecovirus vaccines.

## Seeking broad protection

As scientists develop therapeutic antibodies and vaccines against severe acute respiratory syndrome coronavirus 2 (SARS-CoV-2), the risk of emergent coronaviruses makes it important to also identify broadly protective antibodies. Wec *et al.* isolated and characterized hundreds of antibodies against the viral spike protein of SARS-CoV-2 from the memory B cells of a survivor of the 2003 outbreak caused by the related coronavirus, SARS-CoV. In both of these viruses, the spike protein facilitated viral entry by binding to the angiotensin-converting enzyme 2 (ACE2) receptor on human cells. The antibodies targeted multiple sites on the spike protein, but of nine antibodies that showed strong cross-neutralization, eight targeted the domain that binds to ACE2. These eight antibodies also neutralized a bat SARS-related virus. Illuminating the epitopes on the viral spike protein that bind cross-neutralizing antibodies could guide the design of broadly protective vaccines.

*Science*, this issue p. 731

## Teaser

Broadly neutralizing antibodies that target overlapping epitopes on the SARS-CoV-2 spike protein have been isolated.

In December 2019, a novel pathogen emerged in the city of Wuhan in China’s Hubei province, causing an outbreak of atypical pneumonia [a disease known as coronavirus disease 2019 (COVID-19)]. The infectious agent was characterized as a lineage B betacoronavirus, named severe acute respiratory syndrome coronavirus 2 (SARS-CoV-2) and shown to be closely related to SARS-CoV and several SARS-like bat CoVs (*1*). There are currently no approved vaccines or therapeutics available for the prevention or treatment of COVID-19.

CoV entry into host cells is mediated by the viral S glycoprotein, which forms trimeric spikes on the viral surface (*2*). Each monomer in the trimeric S assembly is a heterodimer of S1 and S2 subunits. The S1 subunit is composed of four domains: an N-terminal domain (NTD), a C-terminal domain (CTD), and subdomains I and II (*3*–*5*). The CTD of both SARS-CoV and SARS-CoV-2 functions as the receptor-binding domain (RBD) for the shared entry receptor, human angiotensin-converting enzyme 2 (hACE2) (*6*–*10*). The S2 subunit contains the fusion peptide, heptad repeats 1 and 2, and a transmembrane domain, all of which are required for fusion of the viral and host cell membranes.

The S glycoprotein of HCoVs is the primary target for neutralizing antibodies (nAbs) (*11*). SARS-CoV and SARS-CoV-2 share 76% amino acid identity in their S proteins, raising the possibility of conserved immunogenic surfaces on these antigens. Studies of convalescent sera and a limited number of monoclonal antibodies (mAbs) have revealed limited to no cross-neutralizing activity, demonstrating that conserved antigenic sites are rarely targeted by nAbs (*5*, *9*, *12*, *13*). However, the frequencies, specificities, and functional activities of cross-reactive antibodies induced by natural SARS-CoV and SARS-CoV-2 infection remain poorly defined.

**Fig. 1:**
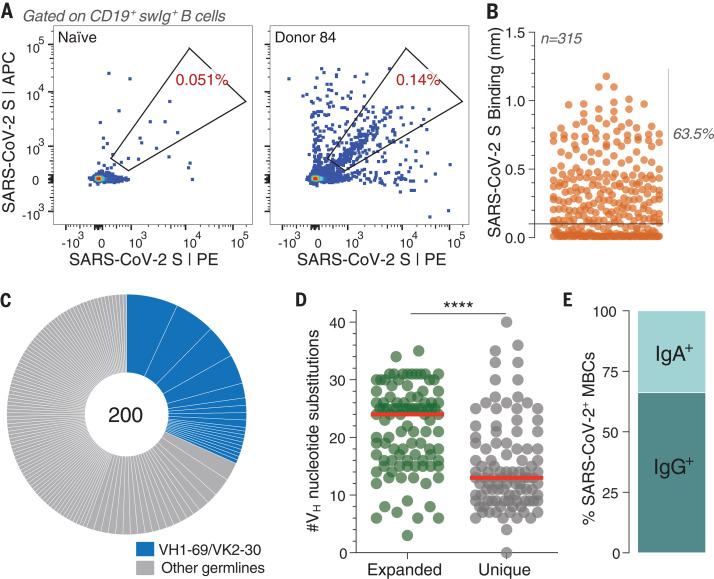
Isolation of SARS-CoV-2 S-specific antibodies. (**A**) Frequency of SARS-CoV-2 S-reactive B cells in donor 84 and a SARS-CoV–naïve donor. Fluorescence-activated cell sorting plots are gated on CD19^+^CD20^+^IgD^−^IgM^−^ B cells. swIg, switched immunoglobulin. (**B**) Binding of 315 isolated antibodies to SARS-CoV-2 S, as determined by BLI. The dashed line indicates the threshold for designating binders (0.1 nm). (**C**) Clonal lineage analysis. Each lineage is represented as a segment proportional to the lineage size. The total number of antibodies is shown in the center of the pie. Clonal lineages were defined based on the following criteria: identical V_H_ and V_L_ germline genes, identical CDR H3 (third complementarity-determining region of the heavy chain) length, and CDR H3 amino acid identity ≥80%. (**D**) Somatic mutation load, expressed as the number of nucleotide substitutions in V_H_, in unique antibodies and members of expanded clonal lineages. Red bars indicate medians. Statistical comparisons were made using the Mann-Whitney test (****P < 0.0001). (**E**) Proportion of SARS-CoV-2 S binders derived from IgG^+^ and IgA^+^ B cells, as determined by index sorting.

We aimed to comprehensively profile the cross-reactive B cell response induced by SARS-CoV infection by cloning an extensive panel of SARS-CoV-2 S-reactive mAbs from the peripheral B cells of a convalescent donor (donor 84) who survived the 2003 SARS outbreak. To isolate cross-reactive antibodies, we obtained a blood sample from this donor about 3 years after infection and stained purified B cells with a panel of memory B cell (MBC) markers and a fluorescently labeled SARS-CoV-2 S protein. Flow cytometric analysis revealed that 0.14% of class-switched MBCs were SARS-CoV-2 S-reactive, which was about threefold greater than background staining observed with a SARS-CoV–naïve donor sample (Fig. 1A). Cognate antibody heavy- and light-chain pairs were amplified from 315 individual SARS-CoV-2–reactive B cells by single-cell reverse transcription polymerase chain reaction (RT-PCR) and subsequently cloned and expressed as full-length immunoglobulin Gs (IgGs) in an engineered strain of *Saccharomyces cerevisiae* (*14*). Of the 315 cloned antibodies, 200 bound to SARS-CoV-2 S in preliminary binding screens (Fig. 1B). Sequence analysis revealed that about half of the clones were members of expanded clonal lineages, whereas the other half were unique (Fig. 1C). Moreover, about 30% of isolated antibodies displayed convergent VH1-69/VK2-30 germline gene pairing (Fig. 1C). As expected, almost all the antibodies were somatically mutated, with members of clonally expanded lineages showing significantly higher levels of somatic hypermutation (SHM) compared with unique clones (Fig. 1D). Finally, consistent with the respiratory nature of SARS-CoV infection, index sorting analysis revealed that 33% of binding antibodies originated from IgA^+^ MBCs and the remaining 66% from IgG^+^ MBCs (Fig. 1E). We conclude that SARS-CoV infection elicited a high frequency of long-lived, cross-reactive MBCs in this donor.

**Fig. 2:**
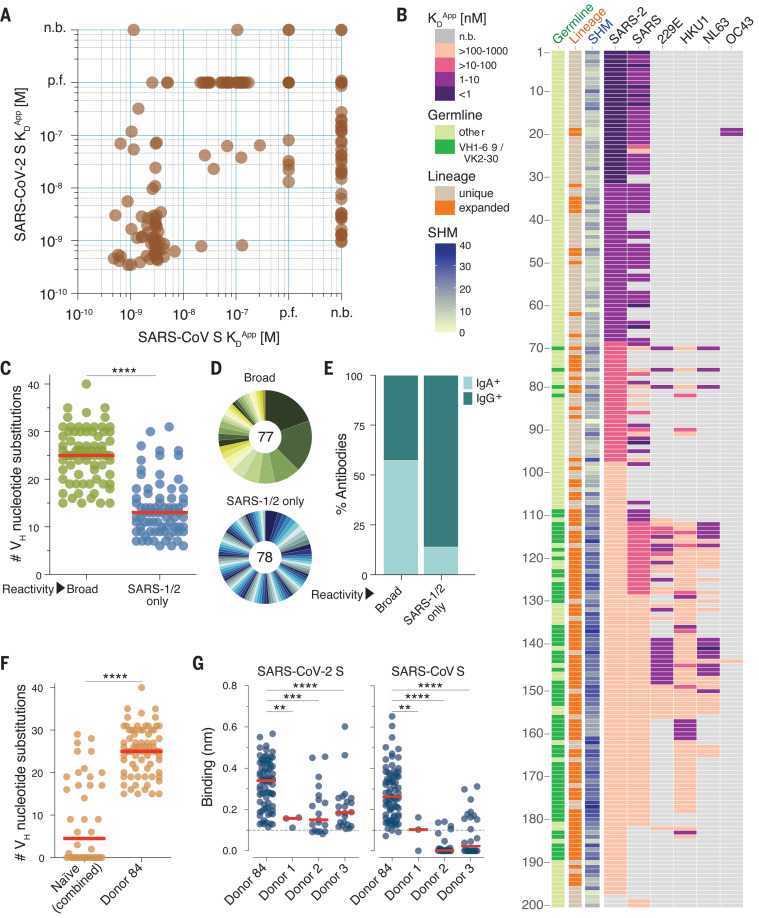
Binding properties of SARS-CoV-2 S-specific antibodies. (**A**) Apparent binding affinities (*K*_D_^Apps^) of SARS-CoV-2 S-specific IgGs for prefusion-stabilized SARS-CoV and SARS-CoV-2 S proteins, as determined by BLI. Low-affinity clones for which binding curves could not be fit are designated as “poor fit” (p.f.) on the plot. n.b., nonbinder. (**B**) IgG *K*_D_^Apps^ for SARS-CoV-2, SARS-CoV, 229E, HKU1, NL63, and OC43 S proteins. Germline gene usage, clonality, and SHM are presented in the three leftmost columns. SHM load is represented as the number of nucleotide substitutions in V_H_. (**C**) Load of somatic mutations in broadly cross-reactive and SARS-CoV– and SARS-CoV-2–specific antibodies. Red bars indicate medians. (**D**) Degree of clonal expansion in broadly cross-reactive and SARS-CoV– and SARS-CoV-2–specific antibodies. Each lineage is represented as a segment proportional to the lineage size. The total number of antibodies is shown in the center of the pie. (**E**) Proportion of broadly cross-reactive and SARS-CoV– and SARS-CoV-2–specific antibodies derived from IgG^+^ and IgA^+^ B cells, as determined by index sorting. (**F**) Load of somatic mutations in SARS-CoV-2 S-reactive antibodies isolated from three naïve donors and donor 84. Antibodies from healthy donors were combined for this analysis. (**G**) Binding activity of antibodies isolated from SARS-CoV-2 S-reactive B cells in donor 84 and three naïve donors to SARS-CoV and SARS-CoV-2 S proteins, as determined by BLI. Statistical comparisons were made using the Mann-Whitney test (**P < 0.01; ***P < 0.001; ****P < 0.0001).

We next measured the apparent binding affinities (*K*_D_^App^s) of the antibodies to prefusion-stabilized SARS-CoV and SARS-CoV-2 S proteins (*5*). Although most antibodies (153 out of 200) showed binding to both S proteins, a subset appeared to be SARS-CoV-2 S-specific (Fig. 2A). This result was unexpected given that the antibodies were isolated from a SARS-CoV–experienced donor and may relate to differences between the infecting SARS-CoV strain and the recombinant SARS-CoV S protein (Tor2) used for the binding studies. Alternatively, this result may be due to inherent differences in the stability or antigenicity of recombinant prefusion-stabilized SARS-CoV and SARS-CoV-2 S proteins. Indeed, about 30% of antibodies that failed to bind recombinant SARS-CoV S displayed reactivity with SARS-CoV S expressed on the surface of transfected cells, providing some evidence for differences in the antigenicity of recombinant and cell-expressed forms of S (fig. S1).

Paradoxically, most of the highly mutated and clonally expanded antibodies bound weakly (*K*_D_^Apps^ > 10 nM) to both SARS-CoV and SARS-CoV-2 S (Fig. 2B). We sought to determine if these antibodies originated from preexisting MBCs induced by prior exposures to naturally circulating HCoVs, which share up to 32% S amino acid identity with SARS-CoV and SARS-CoV-2. Accordingly, we assessed binding of the antibodies to recombinant S proteins of naturally circulating human alphacoronaviruses (HCoV-NL63 and HCoV-229E) and betacoronaviruses (HCoV-OC43 and HCoV-HKU1). More than 80% of the low-affinity (*K*_D_^Apps^ > 10 nM) SARS-CoV and SARS-CoV-2 cross-reactive antibodies reacted with one or more of the HCoV S proteins, suggesting that SARS-CoV infection may have boosted a preexisting MBC response induced by circulating HCoVs (Fig. 2B). Consistent with this hypothesis, the broadly cross-reactive antibodies showed significantly higher levels of SHM and clonal expansion compared with those that only recognized SARS-CoV and SARS-CoV-2 (Fig. 2, B to D). Furthermore, 72% of the broadly binding antibodies used VH1-69/VK2-30 germline gene pairing, suggesting germline-mediated recognition of a common antigenic site (Fig. 2B and fig. S2). Although we were unable to finely map the epitopes recognized by these antibodies, none of them bound to recombinant SARS-CoV-2 S1, suggesting that they likely target epitopes within the more conserved S2 subunit (fig. S3). Index sorting analysis revealed that the majority of the broadly cross-reactive antibodies were derived from IgA^+^ MBCs, indicating a mucosal origin, whereas most of the SARS-CoV and SARS-CoV-2 cross-reactive antibodies originated from IgG^+^ MBCs (Fig. 2E). Finally, all of the broad binders lacked polyreactivity, demonstrating that their cross-binding is not due to nonspecific cross-reactivity (fig. S4).

To investigate whether the above results were due to an original antigenic sin phenomenon, or rather simply due to avid binding of circulating HCoV-specific B cell receptors to the SARS-CoV-2 S tetramers used for cell sorting, we assessed whether similarly broadly binding antibodies were also present in SARS-CoV– and SARS-CoV-2–naïve donors that had been exposed to endemic HCoVs. We obtained peripheral blood mononuclear cell (PBMC) samples from three healthy adult donors with serological evidence of circulating HCoV exposure and no history of SARS-CoV or SARS-CoV-2 infection and stained the corresponding B cells with a fluorescently labeled SARS-CoV-2 S probe (fig. S5A). Flow cytometric analysis revealed that between 0.06 and 0.12% of total B cells in the three naïve donors displayed SARS-CoV-2 reactivity (fig. S5B). More than 350 SARS-CoV-2–reactive MBCs were sorted and amplified by single-cell RT-PCR, and 141 variable region of Ig heavy chain (V_H_)–variable region of Ig light chain (V_L_) pairs were cloned and expressed as full-length IgGs. Although a limited number of SARS-CoV-2 S binding antibodies (3 to 22) were isolated from all three naïve donors, they displayed significantly lower levels of SHM, clonal expansion, and *K*_D_^Apps^ for both SARS-CoV and SARS-CoV-2 S compared with the cross-reactive antibodies identified from donor 84 (Fig. 2, F and G, and fig. S5C). Altogether, these results suggest that SARS-CoV infection likely led to the activation and expansion of preexisting cross-reactive MBCs induced by circulating HCoV exposure in this donor.

**Fig. 3:**
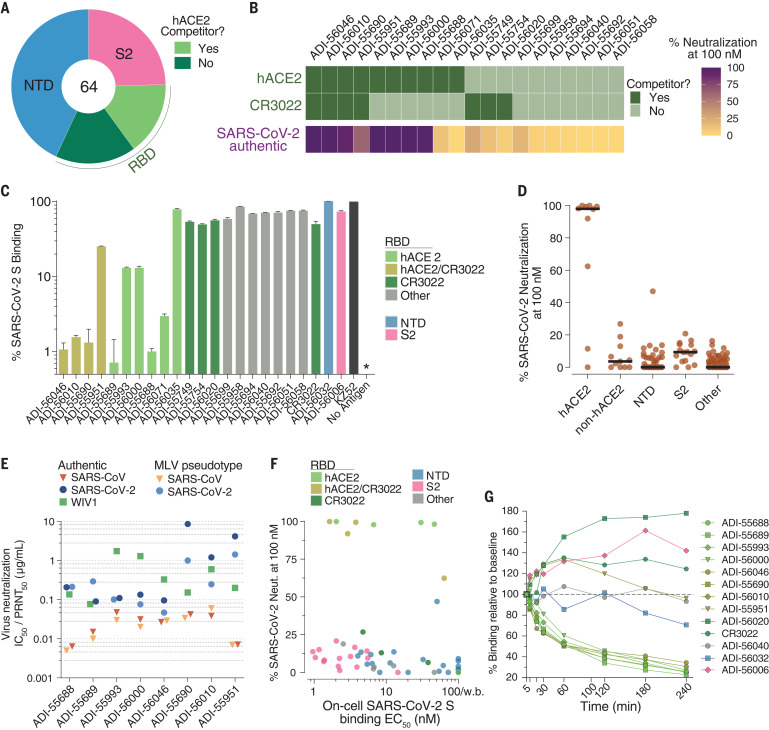
Epitope mapping and neutralization screening. (**A**) Proportion of SARS-CoV-2 S-specific antibodies targeting each of the indicated antigenic sites. (**B**) Heat map showing the competitive binding profiles of the RBD-directed antibodies, as determined by BLI (top) and percent neutralization of authentic SARS-CoV-2 at a 100 nM concentration (bottom). (**C**) Antibody inhibition of SARS-CoV-2 S binding to endogenous ACE2 expressed on Vero E6 cells, as determined by flow cytometry. Antibodies were mixed with recombinant SARS-CoV-2 S bearing a Twin-Strep tag at a molar ratio of 10:1 before adding to Vero E6 cells. An anti-ebolavirus antibody (KZ52) was used as an isotype control. The “no antigen” control indicates secondary-only staining. The asterisk indicates that no detectable binding was observed. Bars are colored according to epitope specificity, as determined in the BLI competition assay. Data represent three technical replicates. (**D**) Percent authentic SARS-CoV-2 neutralization in the presence of 100 nM IgG. Antibodies are grouped according to epitope specificity. RBD-directed antibodies that compete or do not compete with ACE2 are designated as ACE2 and non-ACE2, respectively. (**E**) Antibody neutralization of SARS-CoV and SARS-CoV-2 MLV pseudovirus (strain n-CoV/USA_WA1/2020) using HeLa-ACE2 target cells, and neutralization of authentic SARS-CoV, SARS-CoV-2, and WIV1-CoV using Vero E6 target cells. Data represent two technical replicates. (**F**) Binding EC_50_s for cell-surface SARS-CoV-2 S are plotted against the percent neutralization of authentic SARS-CoV-2 at 100 nM. Background binding was assessed using mock-transfected HEK-293 cells. Data points are colored according to epitope specificity. RBD-directed antibodies are further categorized based on their competition group: hACE2 indicates hACE2-only competitors; CR3022 indicates CR3022-only competitors; hACE2/CR3022 indicates antibodies that compete with hACE2 and CR3022; other indicates hACE2 and CR3022 noncompetitors. Antibodies with cell-binding EC_50_s >100 nM are designated as weak binders (w.b.) on the plot. (**G**) Antibody binding activity to cell-surface SARS-CoV-2 S over time, as determined by flow cytometry. IgGs were incubated with cells expressing WT SARS-CoV-2 over the indicated time intervals. Binding MFI was assessed at 240 min for all samples. CR3022 is included for comparison. Curves are colored by epitope specificity, as in (F). Data represent two technical replicates.

To map the antigenic sites recognized by the SARS-CoV and SARS-CoV-2 cross-reactive antibodies isolated from donor 84, we performed binding experiments using a panel of recombinant S protein subunits and individual domains. Because of the inherent technical challenges associated with measuring binding of low-affinity antibodies to monomeric proteins, we analyzed only the 64 high-affinity binders (*K*_D_^Apps^ < 10 nM) to SARS-CoV-2 S (Fig. 2, A and B). We first evaluated binding to recombinant SARS-CoV-2 S1 and S2 subunits and observed that 75% of the antibodies recognized epitopes within S1, whereas the remaining 25% bound to epitopes within S2 (Fig. 3A). Two of the S2-directed antibodies also showed strong reactivity with OC43 S, suggesting recognition of a conserved antigenic site (fig. S6). We next evaluated the 49 S1-directed antibodies for reactivity with individual SARS-CoV-2 RBD and NTD proteins and found that 21 (43%) and 28 (57%) of the S1-specific antibodies recognized the RBD and NTD, respectively (Fig. 3A).

To further define the epitopes recognized by the 21 RBD-directed antibodies, we performed competitive binding studies with recombinant hACE2 and a previously described antibody, CR3022, that targets a conserved epitope that is distinct from the receptor binding site (Fig. 3B and fig. S7) (*15*). Six of the antibodies competed only with hACE2, three competed only with CR3022, four competed with both hACE2 and CR3022, and seven did not compete with hACE2 or CR3022 (Fig. 3B). Thus, these antibodies delineate at least four adjacent and potentially overlapping sites within the RBD. Most of the antibodies that competed with recombinant hACE2 binding to SARS-CoV-2 RBD in the biolayer interferometry (BLI) assay also interfered with binding of full-length SARS-CoV-2 S to endogenous ACE2 expressed on the surface of Vero E6 cells (Fig. 3C). The four antibodies (ADI-55951, ADI-55993, ADI-56000, and ADI-56035) that showed stronger competition in the BLI assay displayed weak binding affinities for SARS-CoV-2 S (fig. S12), which likely explains their lower level of competition in the cell-surface assay. Thus, SARS-CoV infection elicited high-affinity cross-reactive antibodies to a range of antigenic sites within both the S1 and S2 subunits.

To evaluate the neutralization activities of the SARS-CoV-2 binding antibodies, we performed neutralization assays using both murine leukemia virus (MLV)– and vesicular stomatitis virus (VSV)–based pseudotype systems as well as authentic SARS-CoV-2. Because of the large number of antibodies, we first measured infection inhibition of authentic SARS-CoV-2 at a single concentration of purified IgG. Only 9 out of 200 antibodies displayed neutralizing activity at the 100 nM concentration tested, eight targeted the RBD, and the remaining one recognized the NTD (Fig. 3D). Similar results were observed in the VSV-based pseudovirus assay (fig. S8). Of the eight RBD-directed nAbs, four targeted epitopes overlapping with both the hACE2 and CR3022 epitopes and the other four recognized epitopes overlapping only the hACE2 epitope, suggesting the existence of two partially overlapping neutralizing epitopes within the RBD (Fig. 3B). Neutralization titration studies revealed that the median inhibitory concentrations (IC_50_s) of the RBD-directed nAbs ranged from 0.05 to 1.4 μg/ml against SARS-CoV-2 and 0.004 to 0.06 μg/ml against SARS-CoV in the MLV assay (Fig. 3E and fig. S9). Comparable neutralization IC_50_s were observed in authentic SARS-CoV and SARS-CoV-2 neutralization assays (Fig. 3E and fig. S9). By contrast, the VSV–SARS-CoV-2 neutralization IC_50_s were substantially lower (8- to 35-fold) than those observed for live SARS-CoV-2 (figs. S9 and S10). To assess the breadth of neutralization against representative preemergent SARS-like bat CoVs, we measured infection inhibition of authentic WIV1-CoV using a plaque reduction assay (*16*). All eight antibodies neutralized WIV1-CoV, with median plaque reduction neutralization titers (PRNT_50_s) ranging from 0.076 to 1.7 μg/ml, demonstrating their breadth of activity (Fig. 3E and fig. S11). Crucially, none of the antibodies left an unneutralized viral fraction in any of the assays (figs. S9 and S11).

We observed little to no correlation between apparent binding affinity for wild-type (WT) SARS-CoV-2 cell surface S and neutralizing activity. For example, all of the S2-directed antibodies and a subset of NTD-directed antibodies bound with high avidity to both recombinant and cell surface S, but none were neutralizing (Fig. 3F). Surprisingly, even within the group of hACE2-blocking nAbs, we did not observe a strong correlation between binding to cell surface–S or recombinant-S and neutralization, suggesting that antibody potency is governed at least in part by factors beyond binding affinity (Fig. 3F and figs. S12 and S13). To determine whether the hACE2 competitor antibodies neutralized by inducing S1 shedding and premature S triggering (*17*), we incubated human embryonic kidney (HEK)–293 cells expressing WT SARS-CoV-2 S with saturating concentrations of antibody and measured the median fluorescence intensity (MFI) of antibody binding over time by flow cytometry. Indeed, all of the hACE2-blocking antibodies showed substantially decreased binding over time, consistent with induced S1 dissociation, whereas antibodies recognizing the NTD, S2 stem, and RBD epitopes outside of the hACE2 binding site displayed either no change or an increase in binding over time (Fig. 3G). We conclude that SARS-CoV infection induces high-affinity cross-reactive antibodies targeting multiple distinct antigenic sites on the S protein, but neutralizing activity is primarily restricted to RBD-directed antibodies that interfere with receptor binding and promote S1 dissociation.

**Fig. 4:**
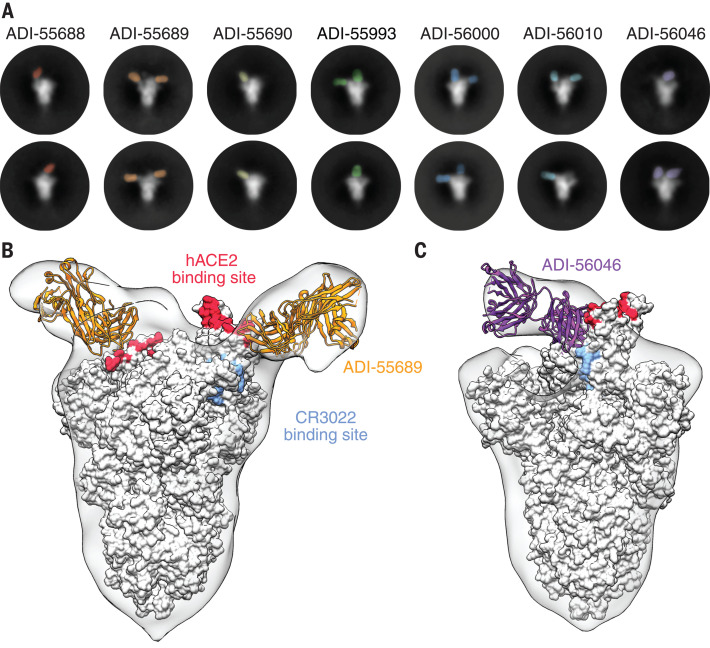
Structures of cross-neutralizing antibodies bound to SARS-CoV-2 S. (**A**) Negative-stain EM 2D class averages of SARS-CoV-2 S bound by Fabs of indicated antibodies. The Fabs have been pseudocolored for ease of visualization. (**B** and **C**) 3D reconstructions of Fab:SARS-CoV-2 S complexes are shown in transparent surface representation (light gray) with the structure of the SARS-CoV-2 S trimer (white surface) and Fabs (ribbon) docked into the density. S-bound Fabs of ADI-55689 (B) and ADI-56046 (C) are colored in orange and purple, respectively. The hACE2 and CR3022 binding sites on S are shaded in red and light blue, respectively.

To structurally characterize the epitopes recognized by the RBD-directed nAbs, we performed negative-stain electron microscopy (EM) to observe each of these Fabs bound to the SARS-CoV-2 S protein. Many of the two-dimensional (2D) class averages that we obtained displayed obvious heterogeneity in the number of Fabs that were bound to a single S trimer, which is likely due to dynamic inaccessibility of RBD epitopes and substoichiometric binding of S at the low protein concentrations used to prepare grids (Fig. 4A) (*5*, *18*). The 3D reconstructions of these complexes support the results of our biophysical competition assays and show that the RBD-directed nAbs recognize a single region on the solvent-exposed surface of the RBD with overlapping footprints. ADI-55689, which potently neutralizes and competes with hACE2, appears to bind at the edge of the hACE2 binding site, close to the more structurally conserved core domain of the RBD, without overlapping with the CR3022 epitope (Fig. 4B). ADI-56046, which exemplifies the group of antibodies that compete with both hACE2 and CR3022, binds slightly farther away from the flexible tip of the RBD, and thus its epitope spans both the hACE2 binding site and the CR3022 epitope (Fig. 4C). Our structural analysis suggests that all of the nAbs recognize a single patch on the surface of the RBD with overlapping footprints. These antibodies potently cross-neutralize SARS-CoV, SARS-CoV-2, and WIV1, suggesting that this antigenic surface exhibits extensive conservation among the SARS-like coronaviruses.

The potent cross-neutralizing antibodies described here bind to conserved epitopes overlapping the hACE2 binding site, thus illuminating this antigenic surface as a promising target for the rational design of pan-sarbecovirus vaccines. For example, the RBD epitope(s) defined by this class of antibodies could be presented on conformationally stable protein scaffolds to focus the antibody response on this site, as previously demonstrated for the motavizumab epitope on respiratory syncytial virus F (*19*). Furthermore, the nAbs themselves, alone or in combination, represent promising candidates for prophylaxis or therapy of SARS, COVID-19, and potentially future diseases caused by new emerging SARS-like viruses.

## Supplementary Materials

science.sciencemag.org/content/369/6504/731/suppl/DC1 Materials and Methods Figs. S1 to S13 References (*20*–*26*) MDAR Reproducibility Checklist

## Appendix

View/request a protocol for this paper from *Bio-protocol*.
